# An Integrated Approach to Develop a Potent Vaccine Candidate Construct Against Prostate Cancer by Utilizing Machine Learning and Bioinformatics

**DOI:** 10.1002/cnr2.70079

**Published:** 2024-12-09

**Authors:** Aqel Albutti

**Affiliations:** ^1^ Department of Basic Health Sciences, College of Applied Medical Sciences Qassim University Buraydah Saudi Arabia

**Keywords:** binding energies, hydrogen bonds, immunoinformatics, prostate cancer, simulation studies

## Abstract

**Background:**

Prostate cancer is the most common malignancy among males. Prostaglandin G/H synthase (PGHS) is an essential enzyme in the synthesis of prostaglandins, and its activation has been linked to many malignancies, including colorectal cancer.

**Aims:**

Due to the limited effectiveness and specificity of existing prostate cancer therapies, this study was designed to formulate improved treatment techniques.

**Methods:**

Several immunoinformatic, reverse vaccinology, and molecular modeling methodologies were used to discover B‐ and T‐cell epitopes for the glioblastoma multiforme tumor PGH2_HUMAN. This research evaluated Prostaglandin G/H synthase 2 protein as a potential vaccine candidate against the malignancy. The multi‐epitope vaccine architecture is engineered to activate the immune system, with each epitope docked to its respective HLAs. Further, MD simulations analysis was performed to validate the findings.

**Results:**

A multi‐epitope subunit vaccine candidate was developed by concatenating the chosen B‐ and T‐cell epitopes. Results yield a codon adaptive index (CAI) of 0.93 and a GC content of 56.77%. Thus, it conforms to a biological requirement for effective protein expression, suggesting competent vaccine efficacy inside the Escherichia coli system. Significant interleukin and cytokine responses were seen, characterized by elevated levels of IL‐2 and IFN‐γ in the immune system's response to the immunization. Molecular docking demonstrated an efficient binding affinity of −278 kcal/mol, with hydrogen bonding to several residues. Furthermore, the system total root mean square deviation (RMSD) reached 3.23 Å, with a maximum of up to 5.0 Å at the 100 ns time point but remains stable till 400 ns time intervals followed by stable root mean square fluctuation (RMSF) and radius of gyration values. The hydrogen bond cloud residues are the critical sites that significantly influence the binding energies of MMPBSA and MMGBSA via substantial van der Waals interactions.

**Conclusion:**

It has been determined that these in silico analyses will further augment the comprehension necessary for advancing the creation of targeted therapies for chemotherapeutic cancer treatments.

## 1 Introduction

Globally, prostate cancer (PCa) is currently the second‐leading cause of death for males in the United States and the third most common cause of cancer‐related deaths in men globally [1]. Significant concerns are prompted by the annual increase in the prevalence, incidence, and death rates of PCa [2]. The American Cancer Society reported 31 620 PCa‐related deaths and 174 650 new cases in the US in 2019 [3]. PCa is often identified by many conventional diagnostic methods, including magnetic resonance imaging, tissue biopsies, and biomarker assessment. Radiation therapy or surgical intervention is often provided to individuals with a pre‐metastatic diagnosis [4]. Androgen deprivation therapy, while associated with various adverse effects including metabolic disturbances, bone weakening, cardiovascular issues, gynecomastia, sexual dysfunction, diabetes, and the potential progression of cancer to drug‐resistant forms, is not wholly effective in treating PCa is treated using many therapeutic modalities, including Radium‐223, innovative hormone therapies, chemotherapy, and palliative care [5]. Notwithstanding the existence of various treatments, an increasing number of people assert that the ailment persists as prevalent and untreatable.

Moreover, William Coley launched the first attempts to develop a cancer vaccine as early as 1890. During that time, Coley treated cancer patients using bacterial extracts because the molecular underpinnings of immunology were not fully understood [6]. Later, scientists investigated the use of deactivated entire cancer cells as a vaccine ingredient. However, this approach likewise did not result in the necessary immunogenicity against cancer [7]. To develop an immunostimulatory vaccine that targets different cancer subtypes, it is imperative to identify antigens linked with tumors. Promising strategies have emerged in the field of immunotherapy recently, such as the utilization of bioinformatics databases containing cancer antigens, which are frequently linked to computational epitope predictions to expedite the creation of cancer vaccines [8].

In this case, the research has been conducted using an immunoinformatic technique. A variety of prediction systems and software programs are utilized to determine the antigenic epitope residues of the required protein [9]. Databases and servers provide a wealth of disease‐specific proteomic data in the post‐genomic era. As a result, immunoinformatic becomes a reliable and affordable technique for scanning proteomes to find antigenic epitopes. To lower the high death rate associated with PCa, the primary goals of this research are to identify recognizable antigenic epitopes for B‐ and T‐cell responses and to develop a multi‐epitope subunit vaccine.

To create a multi‐epitope subunit vaccine, prostaglandin H_2_ (PGH2) PCa‐specific antigens have been chosen [10], due to overexpression of antigens in PCa. These PCa‐specific overexpressed antigens are proficient candidates for developing an MEVC [11, 12]. To find B‐ and T‐cell epitopes, particular antigens associated with PCa were thoroughly analyzed. By connecting the discovered common B‐ and T‐cell epitopes from every protein with a particular peptide linker component, a multi‐epitope vaccine element that might trigger humoral and cell‐mediated immunity is created. As a result, the vaccine component must be combined with an effective adjuvant to encourage an improved immune response. The vaccine constituent is once more assessed by VaxiJen analysis to confirm its antigenicity and is necessary for an effective immunogen [13, 14]. Afterward, the three‐dimensional (3D) geometry of the vaccine component is modeled and verified, as molecular docking requires the vaccine's 3D coordinates. Furthermore, utilizing molecular docking experiments, the binding interaction among the receptor protein and vaccine complexes has been evaluated [15]. Followed by molecular dynamic simulation studies stability of the complex system has been monitored in real‐time with thermodynamics and its mechanistic behavior. Hence, the study aims to design a multiepitope subunit vaccine to prevent PCa by employing a variety of immunoinformatic approaches.

## 2 Materials and Methods

**FIGURE 1:**
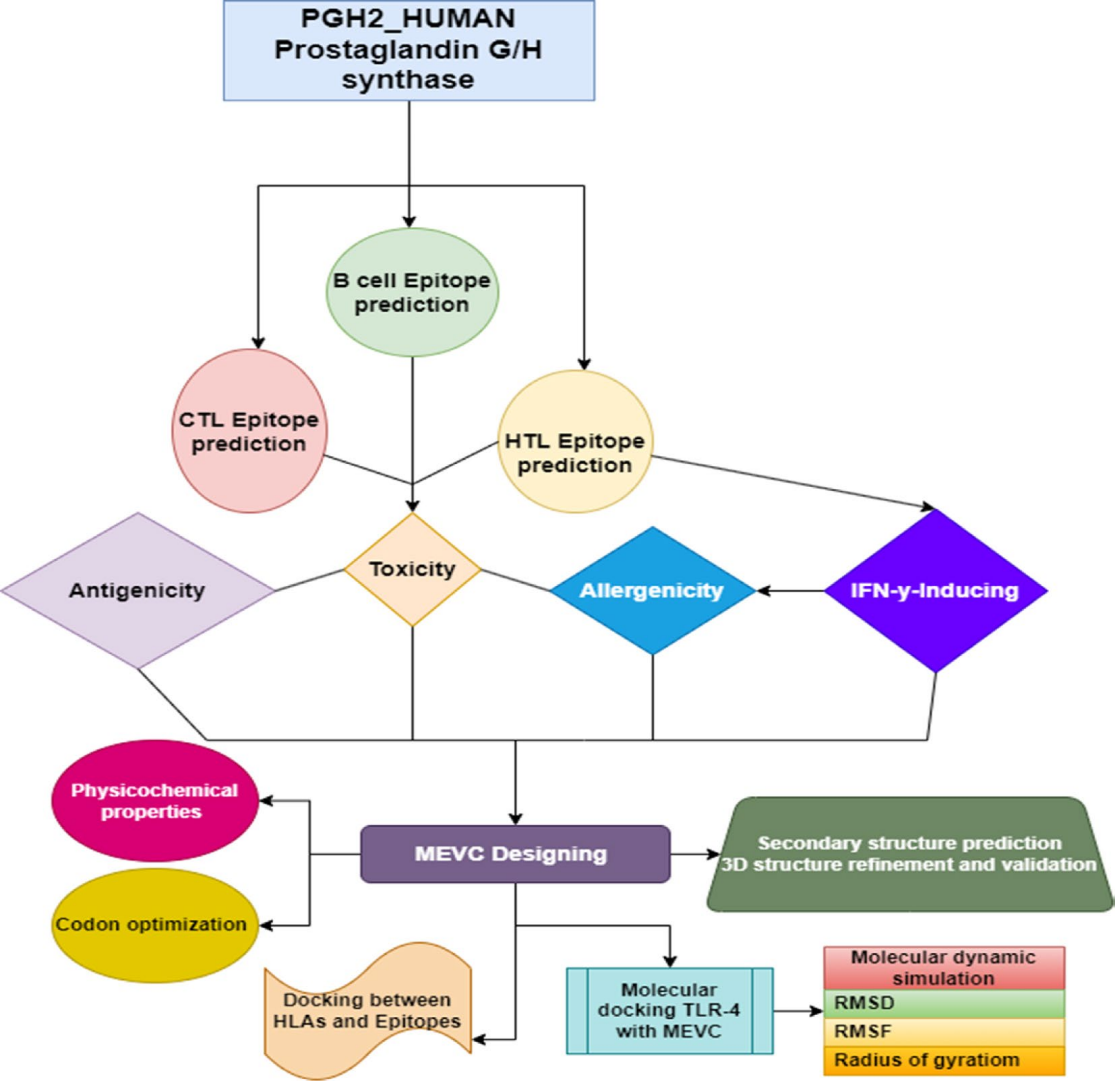
Flow chart depicting the protocol for vaccine designing against PCa.

The current research work is designed to frame the different approaches used during the vaccine design as shown in a flow chart (Figure 1).

### 2.1 Retrieval of Proteomic Data

Proteomic information was acquired from the UniProt database for the target antigen PGH2 linked to PCa (Uniprot ID: P35354⋯PGH2_HUMAN) [16]. The target protein's sequences are meticulously taken out of the NCBI proteome database. After this acquisition, a thorough analysis was performed to pinpoint and define the epitope regions found in these sequences.

### 2.2 T‐Cell Epitope Identification and Assessment

Identifying T‐cell epitopes constitutes a crucial stage in vaccine development. This procedure not only improves cost‐effectiveness but also drastically shortens the process's total duration in comparison to conventional laboratory investigations [17, 18]. The human species was identified as the source, the consensus approach was chosen as the prediction technique, and all submitted alleles were used for epitope prediction [19]. There are many epitopes, thus, to determine which are most appropriate, a screening process is required to evaluate each epitope's antigenicity, toxicity, and allergenicity. The antigenic characteristics of the epitopes were predicted using the VaxiJen v2.0 server (http://www.ddgpharmfac.net/vaxijen/) (accessed in March 2024) [20]. The tumor was chosen as the target organism for this evaluation, and an antigenic characteristic threshold of 0.5 was set. Toxin Pred is an online program [21], that predicts the toxicity of epitopes by applying a quantitative matrix and machine learning. In addition, the AlgPred 2.0 server (accessed in March 2024) was employed to evaluate the allergenic properties of the epitopes [22].

### 2.3 Recognition and Choosing of B‐Cell Epitopes

The successful B‐cell epitope screening validates the immunoinformatic field's strategic development of epitope vaccines [23]. Two different prediction servers, BCPreds and Immune Epitope Database (IEDB) (accessed in March 2024) were used to accurately identify B‐cell epitopes based on the protein sequences of selected prostate antigen [24, 25]. The BCPreds prediction support applied the BCPreds techniques and steps, which are based on amino acid pair antigenicity and string kernel approaches [26]. B‐cell epitope identification using both BCPreds prediction methods. On the other hand, B‐cell epitopes were found using the BepiPred Linear Epitope Prediction 2.0 module in IEDB [27].

### 2.4 Peptide Modeling and Docking Studies

The PEP‐FOLD v3.0 server was used to analyze the chosen Cytotoxic T lymphocytes (CTL) and Helper T lymphocytes (HTL) epitopes to assess the binding efficiency and forecast the peptide structure [28]. 200 sOPEP sorting algorithm simulations were used to achieve it [29]. The Protein Data Bank provided information on the crystal structure of HLA [30]. The docked complex was created and visualized using PyMOL molecular graphic system version 1.3, and docking was performed via HawkDock Server (http://cadd.zju.edu.cn/hawkdock/) (accessed in March 2024) [31].

Moreover, the presence of immunological receptors in immune cells plays a major role in the accuracy of the immune response. Molecular docking was utilized to examine the relationship between the vaccine and the TLR‐4 receptor (PDB ID: 3fxi) using the HDOCK server (http://hdock.phys.hust.edu.cn/) [32], This was carried out in order to evaluate the vaccine's ability to trigger an immune response. Since TLR‐4 is a dimer, interactions with both of its chains were part of the evaluation. The PDBsum server (accessed in March 2024) [33] was used to assess the connection between MEVC and TLR‐4 residues.

### 2.5 Development of the Vaccine Construct

The epitopes identified in the previous step were used to construct a multi‐epitope vaccine. GPGPG linkers were used to connect Helper T‐cell (HTL) epitopes, owing to their shown capacity to preserve the structural integrity and functional distinction of the epitopes [34]. AAY linkers were used to connect Cytotoxic T‐cell (CTL) epitopes. AAY linkers are recognized for their adaptability and efficacy in preserving the proper alignment and display of CTL epitopes, therefore optimizing their immunogenic potential [35, 36]. In addition, an adjuvant DMXAA was used to stimulate interferon‐mediated dendritic cell activation which will then boost the vaccine candidate's immunogenicity [37]. It has a powerful capacity to activate the immune system. DMXAA, a vascular‐disrupting drug, has shown considerable effectiveness in augmenting the immune response by stimulating the generation of pro‐inflammatory cytokines and activating dendritic cells. This leads to a more vigorous and enduring immune response, rendering DMXAA an essential element in attaining the required efficiency of vaccination [38].

### 2.6 Disulfide Engineering

The multi‐epitope vaccine (MEV) build was developed using a disulfide engineering method to enhance structural stability. The disulfide bonds were included in the structure with the bioinformatics program Design2.0 [39].

### 2.7 Physicochemical Evaluation of MEV

Several web‐based tools were used to evaluate the final vaccine structure in terms of its molecular and physical properties, antigenic potential, and allergy potential. The AlgPred 2.0 server (accessed in March 2024) [40] was used to assess the allergenicity of the construct, whereas the VaxiJen v2.0 server was utilized to forecast the antigenic properties of the MEV construct. Furthermore, a program called Expasy Protparam was used to determine the physicochemical properties of the final MEV construct [41]. Determining the physicochemical behavior and expected half‐lives of yeast, *Escherichia coli* , and human cells was required in order to do this [42].

### 2.8 Predication of Protein Secondary and Tertiary Structure

The PSI‐PREDV3.3 server [43] (accessed in March 2024) was used to forecast the secondary structural elements of the build sequence. Various server configurations are employed to achieve extremely precise tertiary structure modeling [44]. Nonetheless, the Robetta server was created for the comparative modeling of 3D protein structures due to this display of target‐template coherence [45]. Furthermore, this 3D structure was improved using the GalaxyRefine web server (https://galaxy.seoklab.org/cgi‐bin/submit.cgi?type=REFINE) (accessed in March 2024), which uses CASP10 to refine the query's 3D structure [46]. The side chains of the protein are recreated using the CASP10 method. After that, the structure is repackaged, and 3D‐structure simulations are used to accomplish relaxation.

### 2.9 Validating the Tertiary Structure of Vaccine

A key initial phase in determining the protein's 3D configuration is to validate the tertiary structure. ProSA‐web, the ERRAT website, and Ramachandran plot evaluation were used in this present study to validate the protein 3D structure [47]. The Z‐score is a quality statistic computed for a specific 3D structure by the ProSA‐web server (accessed in March 2024) [41]. The ERRAT server [48] was used to evaluate and calculate the nonbonded interactions present in the 3D structure. The examination of the Ramachandran plot was facilitated by the PROCHECK server [49]. The 3D vaccine structure was also subjected to prediction of discontinuous B‐cell epitopes using DiscoTope (http://tools.iedb.org/discotope/).

### 2.10 Immuno‐Simulation

Using an agent‐based modeling method, the web‐based server C‐IMMSIM assesses the immune system's response to foreign antigen particles [50]. Using the PSSM approach, the server simulates how the immune system would respond to the antigen. Following immunization, levels of interferon production, cytokines, and antibodies were measured. For most current vaccines, the suggested interval concerning the initial and subsequent doses is 4 weeks [51]. All the parameters were set to their default values, and the time steps were set at intervals of 1, so each time step was equivalent to 8 h. As a result, there was a four‐week gap between the two doses administered. This web server additionally provides Th1 and Th2 response forecasts.

### 2.11 Codon Adaptation for Vaccine Construct

Once the designed MEVC features and in silico immunological simulations were thoroughly analyzed, the Java Codon Adaptation Tool (https://www.jcat.de/) (accessed in April 2024) [52] was used to optimize the codons. The prokaryotic expression system utilized for the MEVC codon optimization was *E. coli* K12. Three possibilities were accessible for identification: [1] prokaryotic ribosome binding sites; [2] restriction enzyme cleavage sites; and [3] rho‐independent transcription termination sites. To ensure high levels of protein expression, the JCat output includes a codon adaptation index (CAI) and GC content [46]. Before being inserted into the pVAX1 vector, the target gene's C‐ and N‐terminals, respectively, were flanked with NotI and XhoI restriction sites. All things considered, the pVAX1vector was incorporated to improve vaccine expression within the altered sequence while taking the specified restriction site restrictions into account.

### 2.12 Population Coverage

The distribution and expression of HLA alleles may vary globally owing to regional and ethnic disparities. Consequently, evaluating epitope population coverage is crucial for ascertaining the effectiveness of MEV s across diverse global populations [53]. A population coverage assessment was conducted for B‐cell, CTL, and HTL epitopes globally using the IEDB's population coverage tool (http://tools.iedb.org/population/) [54].

### 2.13 Molecular Docking

Exposed surface epitopes were docked with the 3D structure of the predominant HLA‐A68‐peptide docking [55]. PEP‐FOLD3 was used to construct the 3D structure of epitopes, using 3D representations of HLAs sourced from the Protein Data Bank (PDB ID: 4hwz). The binding of peptides to major histocompatibility complexes (MHC) complexes was assessed using HLA‐peptide docking by applying the HDOCK server. Whereas, on the other hand, the TLR‐4 receptor was retrieved from the PDB with PDB ID: 3fxi and the optimal complex solution was displayed using UCSF Chimera and LigPlot (accessed in April 2024) [56, 57]. The intricate selection was predicated on the resemblance score, interaction score, and projected accuracy [58, 59]. Furthermore, hydrogen bonding and hydrophobic interactions between the peptide and the active site residues of the receptor protein were also considered [60]. These interactions are crucial for complex stability and were considered to elucidate epitope stability in the receptor's active region [60].

### 2.14 Molecular dynamics (MD) Simulation

MD simulations were performed to observe the dynamic behavior of docked complexes. The AMBER16 program is used to perform simulations followed by consecutive analyses that were made through its different modules [61]. The tleap program of AMBER16 is used to build topology files and missing atoms were added. System solvation was performed with three‐point convertible intermolecular potential (TIP3P) water, while, the force fields engaged for calculations include GAFF [62], and ff14SB [63]. The trajectories of MD simulations were examined through the AMBER PTRAJ module. These analyses are comprised of root mean square deviation (RMSD), root mean square fluctuation (RMSF), radius of gyration (*R*_g_), hydrogen bonding, binding free energies (MM(PB/GB)SA), Waterswap calculation and entropy analysis. For graphical analysis and evaluation of some of these parameters, a two‐dimensional (2D) “Xmgrace” plotting tool is utilized [64].

### 2.15 Interaction Analysis

Interaction analysis plays a vital role in the analysis of any docked complex. It helps to detect the orientation and position of the vaccine construct within the binding site. Furthermore, it helps to define the stability of the complex. The interaction analysis includes both hydrogen and van der Waals interactions. Hydrogen Bonding displays a directionality and interaction specificity between a protein and its ligand (protein, effector, inhibitor, nucleic acid, or substrate) which is a key aspect of molecular recognition [65]. Therefore, the energy and kinetics of hydrogen bonds must be optimal to allow rapid sampling and folding kinetics. This confers stability in the protein structure and specificity required for selective macro‐molecular interactions [65]. The hydrogen bond plot was extracted over time using the cpptraj module of AMBER16. A fraction of ≥ 0.05 Å for donor and acceptor atoms is chosen as a default parameter.

## 3 Results

### 3.1 Retrieval of Proteomic Data

The amino acid sequences of Prostaglandin G/H synthase 2 (PGH2) were retrieved from UniProt, identified by the accession number P35354, constituting a protein of 604 amino acids. Notably, in cases of human PCa, there is a significant upregulation of the expression of these proteins within the prostate tissue. Utilizing VaxiJen v.2.0, an evaluation of the antigenicity of the target protein was conducted, revealing its antigenic properties. To assess the similarity of this protein to human proteins, sequence analysis was performed using Blastp. The results from the Blastp study indicated no apparent similarity between the protein and human proteins. Subsequently, these acquired sequences were employed in predicting T and B‐cell epitopes, contributing to the development of a multi‐epitope subunit vaccine.

### 3.2 Identification and Selection of B‐Cell Epitopes

The outcomes from both prediction servers indicate minimal disparities in the identification of B‐cell epitopes, as summarized in Table 1. Consequently, consensus was established by accepting the shared epitopic sequences identified by all prediction methods, formally designated as B‐cell epitopes (refer to Table 2). Four distinct epitopes from PGH2 were identified and acknowledged as potential B‐cell epitopes and were then subjected to evaluation using VaxiJen v2.0, AlgPred 2.0, and ToxinPred for their antigenic, allergic, and toxic properties.

**TABLE 1:** Epitopes shared between IEDB and BCPreds web server predictions, specifically selected for B cells.

Rank	Sequence	Start position	Score
1	SHLIDSPPTYNADYGY	107	0.97
2	TASIQSLICNNVKGCP	547	0.93
3	AEMIYPPQVPEHLRFA	257	0.92
4	DVLKQEHPEWGDEQLF	300	0.91
5	SPAYWKPSTFGGEVGF	527	0.90
5	ANPCCSHPCQNRGVCM	18	0.90
6	TRQIAGRVAGGRNVPP	413	0.88
7	TVTINASSSRSGLDDI	576	0.87
7	TSFSVPDPELIKTVTI	564	0.87
7	YKCDCTRTGFYGENCS	40	0.87
7	HWHPLLPDTFQIHDQK	372	0.87
7	HPCQNRGVCMSVGFDQ	24	0.87
7	PPVPDDCPTPLGVKGK	139	0.87
7	YYTRALPPVPDDCPTP	133	0.87
8	PALLVEKPRPDAIFGE	491	0.86
8	YQIIDGEMYPPTVKDT	240	0.86
9	YATIWLREHNRVCDVL	287	0.85
9	SWEAFSNLSYYTRALP	124	0.85
10	YGDIDAVELYPALLVE	481	0.84
11	QASIDQSRQMKYQSFN	435	0.83
12	HPEWGDEQLFQTSRLI	306	0.82
12	RKFIPDPQGSNMMFAF	171	0.82
12	PPTYNADYGYKSWEAF	113	0.82
13	LFLKPTPNTVHYILTH	65	0.80
13	APFSLKGLMGNVICSP	513	0.80
13	FEELTGEKEMSAELEA	464	0.80
13	AVLALSHTANPCCSHP	10	0.80
14	YGENCSTPEFLTRIKL	50	0.79
14	QIHDQKYNYQQFIYNN	382	0.79
15	KIVIEDYVQHLSGYHF	328	0.78
16	MGNVICSPAYWKPSTF	521	0.77
16	LSGYHFKLKFDPELLF	338	0.77
17	PNTVHYILTHFKGFWN	71	0.75
17	PSTFGGEVGFQIINTA	533	0.75
18	LILIGETIKIVIEDYV	320	0.74
18	VCMSVGFDQYKCDCTR	31	0.74
19	HIYGETLARQRKLRLF	218	0.73
20	RPDAIFGETMVEVGAP	499	0.71
20	IAAEFNTLYHWHPLLP	363	0.71
20	PPTVKDTQAEMIYPPQ	249	0.71
20	HFTHQFFKTDHKRGPA	190	0.71
21	MSYVLTSRSHLIDSPP	99	0.70
21	KRFMLKPYESFEELTG	454	0.70
22	SRSGLDDINPTVLLKE	584	0.69
22	KTDHKRGPAFTNGLGH	197	0.69
23	QMKYQSFNEYRKRFML	443	0.68
23	EHLRFAVGQEVFGLVP	267	0.68
24	SNEIVEKLLLRRKFIP	160	0.64
24	TPLGVKGKKQLPDSNE	147	0.64
25	GPAFTNGLGHGVDLNH	203	0.62
26	NKQFQYQNRIAAEFNT	354	0.60
27	RVAGGRNVPPAVQKVS	419	0.59
28	GQEVFGLVPGLMMYAT	274	0.58
29	SILLEHGITQFVESFT	398	0.57
30	ILTHFKGFWNVVNNIP	77	0.56
31	PQGSNMMFAFFAQHFT	177	0.53
32	NVVNNIPFLRNAIMSY	86	0.52

**TABLE 2:** Epitopes were chosen for both MHC molecules, targeting both B and T cells.

Epitopes	Score	Antigenicity	Toxicity	Allergenicity
B‐Cell				
PPVPDDCPTPLGVKGK	0.6422	Antigen	Nontoxic	Nonallergen
YQIIDGEMYPPTVKDT	0.6728	Antigen	Nontoxic	Nonallergen
LFLKPTPNTVHYILTH	0.6789	Antigen	Nontoxic	Nonallergen
FEELTGEKEMSAELEA	0.7351	Antigen	Nontoxic	Nonallergen
CTL				
RQRKLRLFK	1.1966	Antigen	Nontoxic	Nonallergen
GENCSTPEF	1.3458	Antigen	Nontoxic	Nonallergen
KYQSFNEYR	0.5970	Antigen	Nontoxic	Nonallergen
EAFSNLSYY	0.7577	Antigen	Nontoxic	Nonallergen
HTL				
KLRLFKDGKMKYQII	1.1209	Antigen	Nontoxic	Nonallergen
GETIKIVIEDYVQHL	0.5197	Antigen	Nontoxic	Nonallergen
MSYVLTSRSHLIDSP	0.6338	Antigen	Nontoxic	Nonallergen
ETIKIVIEDYVQHLS	0.5889	Antigen	Nontoxic	Nonallergen

### 3.3 T‐Cell Epitope Identification Among Selected B‐Cell Epitopes

In the context of T‐cell‐mediated immune responses, the binding of peptides to MHC serves as a critical determinant for specific cellular immunogenicity. This study discloses the identification of eight T‐cell epitopes that interact with both MHC‐I and MHC‐II classes. CTL play a pivotal role in the cellular immune response, recognizing immunogenic antigens on the surfaces of virus‐infected cells. The T‐cell receptor, specific to the antigen, binds to CTL epitopes and MHC class‐I molecules, forming complexes on the surfaces of virus‐infected cells. Consequently, 9‐mer CTL epitopes were predicted, and four potential CTL epitopes were selected targeting the human allele HLA‐A*68:01.

HTL are central to the adaptive immune response, stimulating B cells to secrete antibodies and activating other T cells. In this study, four HTL epitopes were predicted using the NN‐align method from the IEDB server, targeting the human allele HLA‐DRB1*15:01. The predicted epitopes underwent analysis for antigenicity, allergenicity, and toxicity, as detailed in Table 2. All the predicted and final sets of epitopes were revealed to be presented in the predicted discontinuous B‐cell epitopes.

### 3.4 Multi‐Epitopes Vaccine Formulation

**FIGURE 2:**
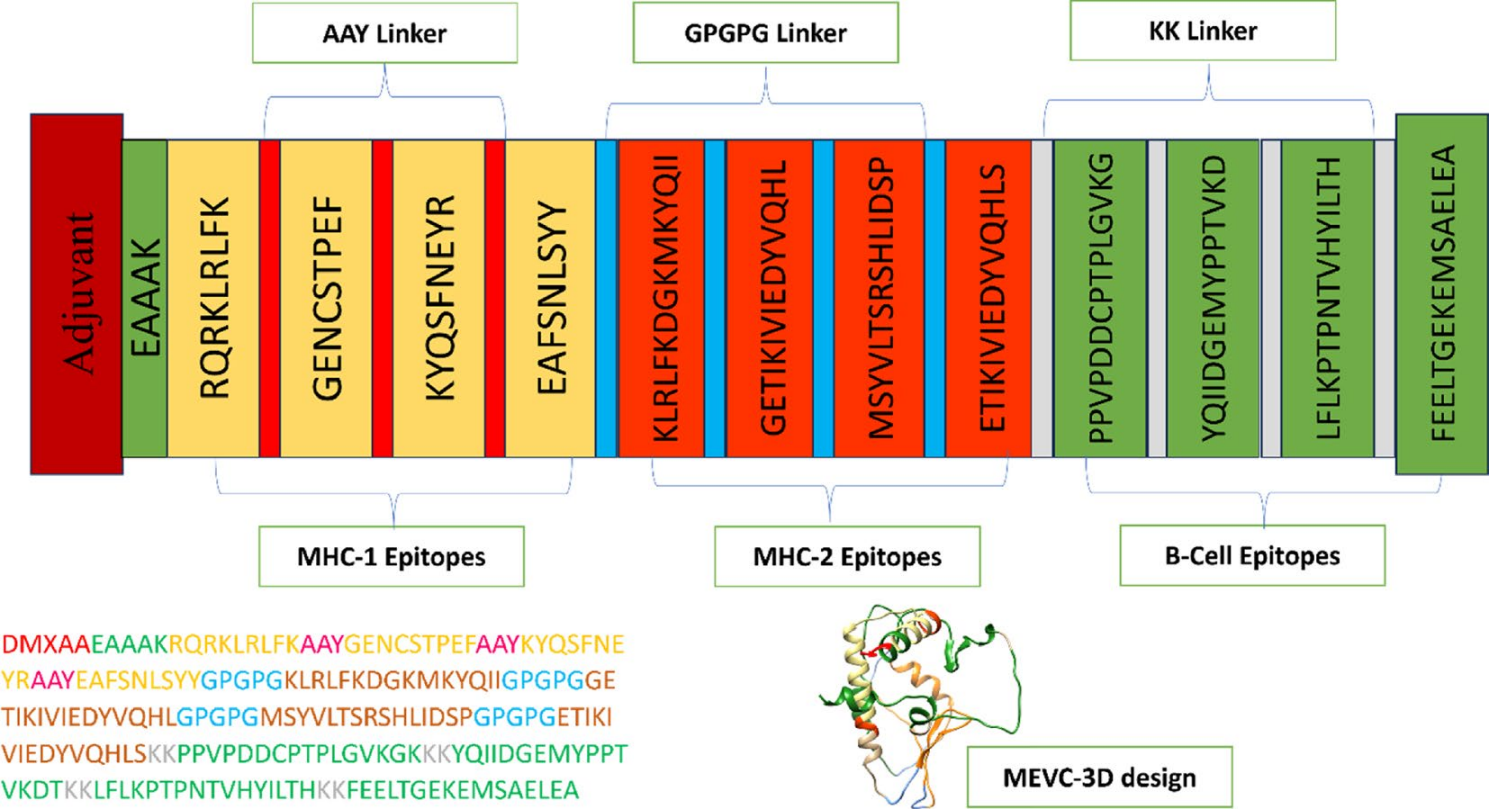
Multi‐epitope construct for PCa. Color code presenting the key domains of the vaccine constructs with 3D modeled design.

The MEV was designed with 12 common B‐ and T‐cell epitopes that were carefully linked together with a GPGPG flexible peptide linker to form a single vaccine protein. This peptide linker has two functions: it increases total biological activity and stabilizes proteins. DMXAA, an agonist of the stimulator of interferon genes (STING), was added to the vaccine formulation as a supplement to promote interferon‐mediated dendritic cell activation. Additionally, DMXAA possesses potent anti‐tumor and anti‐vascularization properties, rendering it an ideal functional adjuvant for a cancer vaccine Figure 2.

### 3.5 AI‐Based Model of Vaccine Construct

The AI‐based 3D model conformation of the vaccine component was achieved using the AlphaFold server. This vaccine element is characterized by the presence of α‐helices and a singular beta‐sheet, while the remaining portion adopts a random coil structure.

### 3.6 Disulfide Engineering

Enhancing protein stability is a primary objective in protein engineering. The most logical strategy is to enhance the stabilizing molecular connections inherent in proteins [39]. Disulfide bridges are covalent connections that provide significant structural stability. Disulfide engineering is a technique for incorporating disulfide connections into the vaccination framework to enhance its structural stability. Numerous residues are susceptible to enzymatic degradation; thus, they are substituted by cysteine residues in the vaccine formulation [66]. The altered residues and the structures of the mutant and original planned vaccine are seen in Figure S2.

### 3.7 Vaccine Model Validation

**FIGURE 3:**
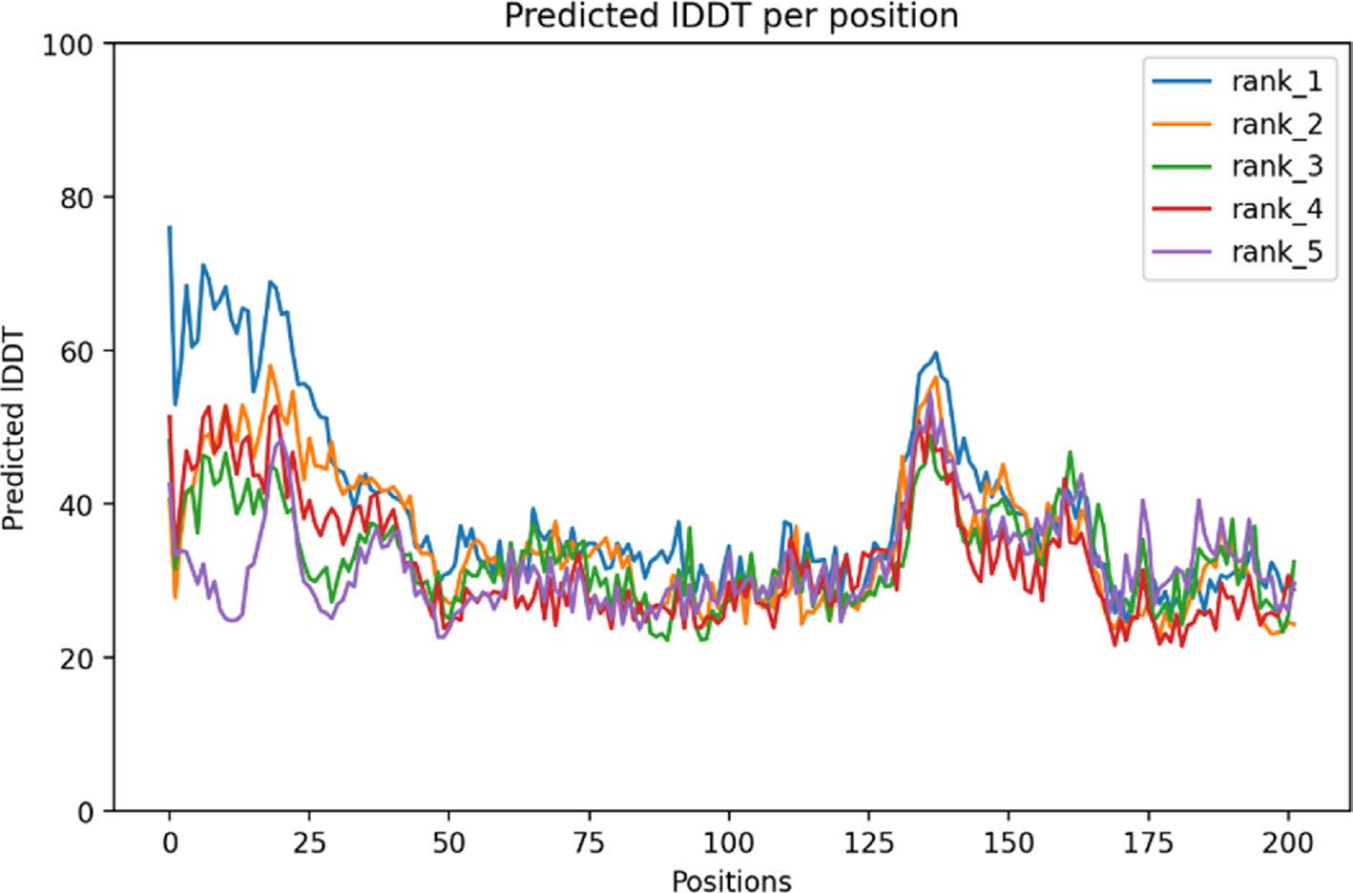
Per‐position plots depict the predicted Inter‐Atomic distance deviation (IDDT) for the top five models, providing insights into their conformational stability.

The AlphaFold tool generates a confidence estimate for each residue, ranging from 0 to 100, denoted as pLDDT. This metric corresponds to the model's predicted score on the lDDT‐Cα scale. It is expected that regions with pLDDT values greater than 90 would show good modeling accuracy. As a sign of a usually reliable prognosis, those with pLDDT levels between 70 and 90 are anticipated to have well‐constructed backbones. Regions with pLDDT values between 50 and 70 are considered low confidence and should be approached with caution. For regions where pLDDT is less than 50, the 3D coordinates may exhibit a ribbon‐like appearance and are not recommended for interpretation. The current study presented in our paper demonstrates that pLDDT values below 50 serve as a reasonably strong indicator of disorder, suggesting either an absence of structure under physiological conditions or structural existence only within a complex. Confident regions typically do not exhibit unphysical bond lengths or clashes. Additionally, a per‐position pLDDT plot (Figure 3) is provided for the five models generated in each run, with the results indicating that model 5 is the most optimal.

**FIGURE 4:**
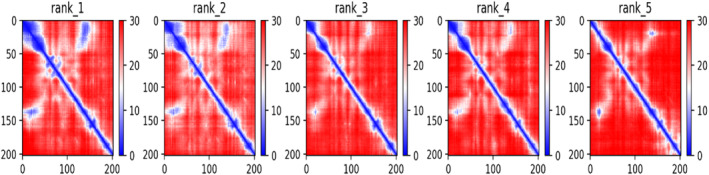
Five models of the vaccine construct were derived from AlphaFold's predictions across a range of structures.

The color at coordinates (*x*, *y*) in this output signifies AlphaFold's anticipated positional error at residue *x*, assuming alignment between the predicted and true structures on residue *y*. When the predicted aligned error is consistently low for residue pairs *x*, *y* originating from distinct domains, it indicates that AlphaFold accurately predicts well‐defined relative positions for these residues. Conversely, if the predicted aligned error is consistently high for residue pairs *x*, *y* from different domains, it implies uncertainty in the relative positions of these domains in the 3D structure, cautioning against definitive interpretations. In a general assessment, the predicted aligned error (PAE) plot exhibits favorable characteristics. Notably, in models 4 and 5, the positioning of the C‐terminus relative to most of the protein is notably superior compared to models 1 to 3 as shown in (Figure 4). Alongside the Ramachandran plot obtained via pdbsum server insights most of the residues in favored region, followed by allowed and disallowed region as shown in Figure S1.

### 3.8 Population Coverage

**FIGURE 5:**
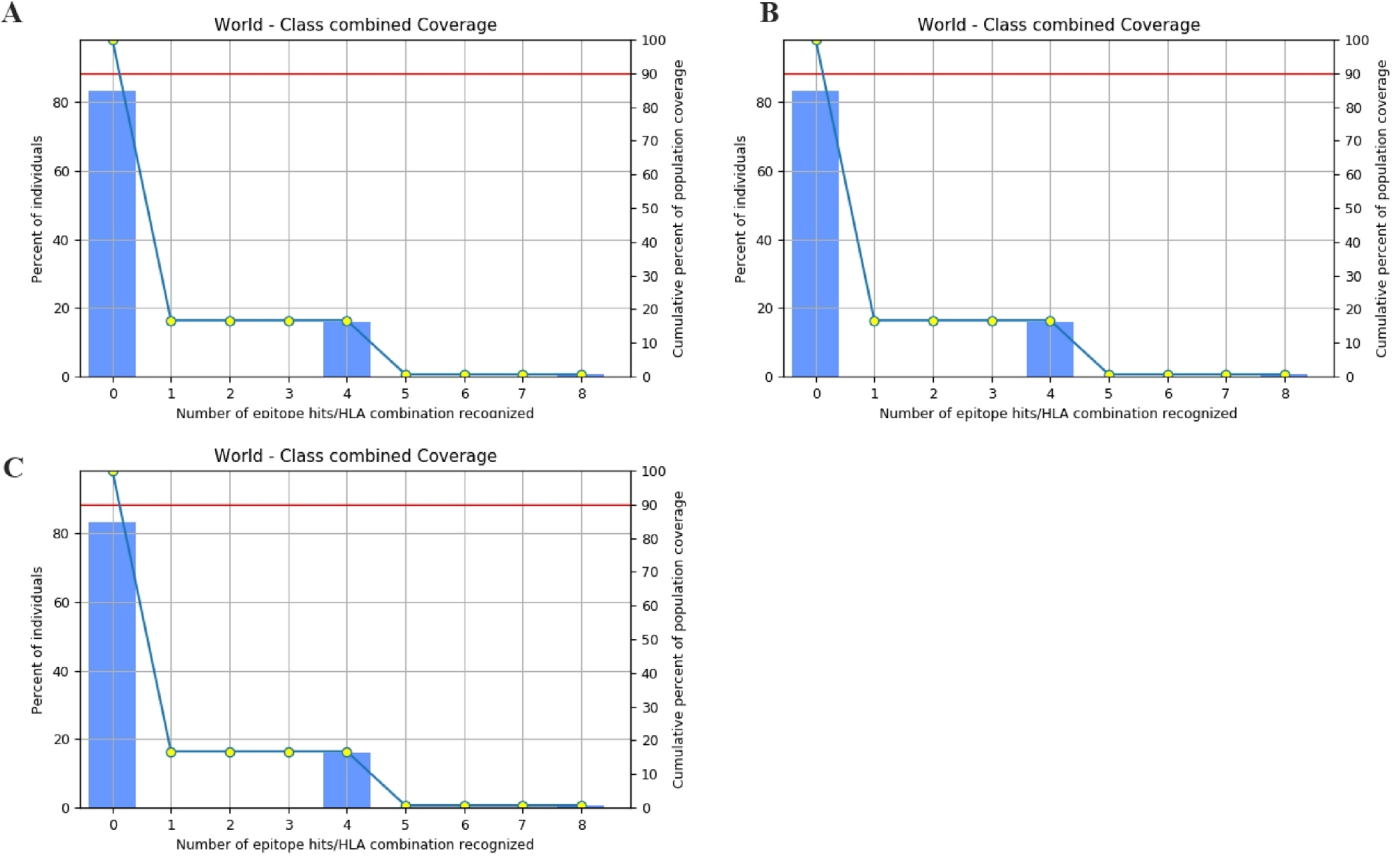
Global population coverage of the identified epitopes for the vaccine design worldwide. The y‐axis represents the nations, while the *x*‐axis denotes the percentage coverage. The population coverage % is shown next to the bars. (A) Depicting the combined population coverage for B‐cell epitope (B) Shows the CTL epitopes population coverage and (C) Illustrates the HTL epitopes population coverage worldwide.

The IEDB anticipated that chosen B‐cell, CTL and HTL epitopes would include 16.7% of the combined global population, respectively as shown in Figure 5. while their amalgamation would encompass an increase in the global population. This indicates that our vaccine, derived from these targeted epitopes, will effectively address this pandemic.

### 3.9 HLA‐Epitope Molecular Docking

**FIGURE 6:**
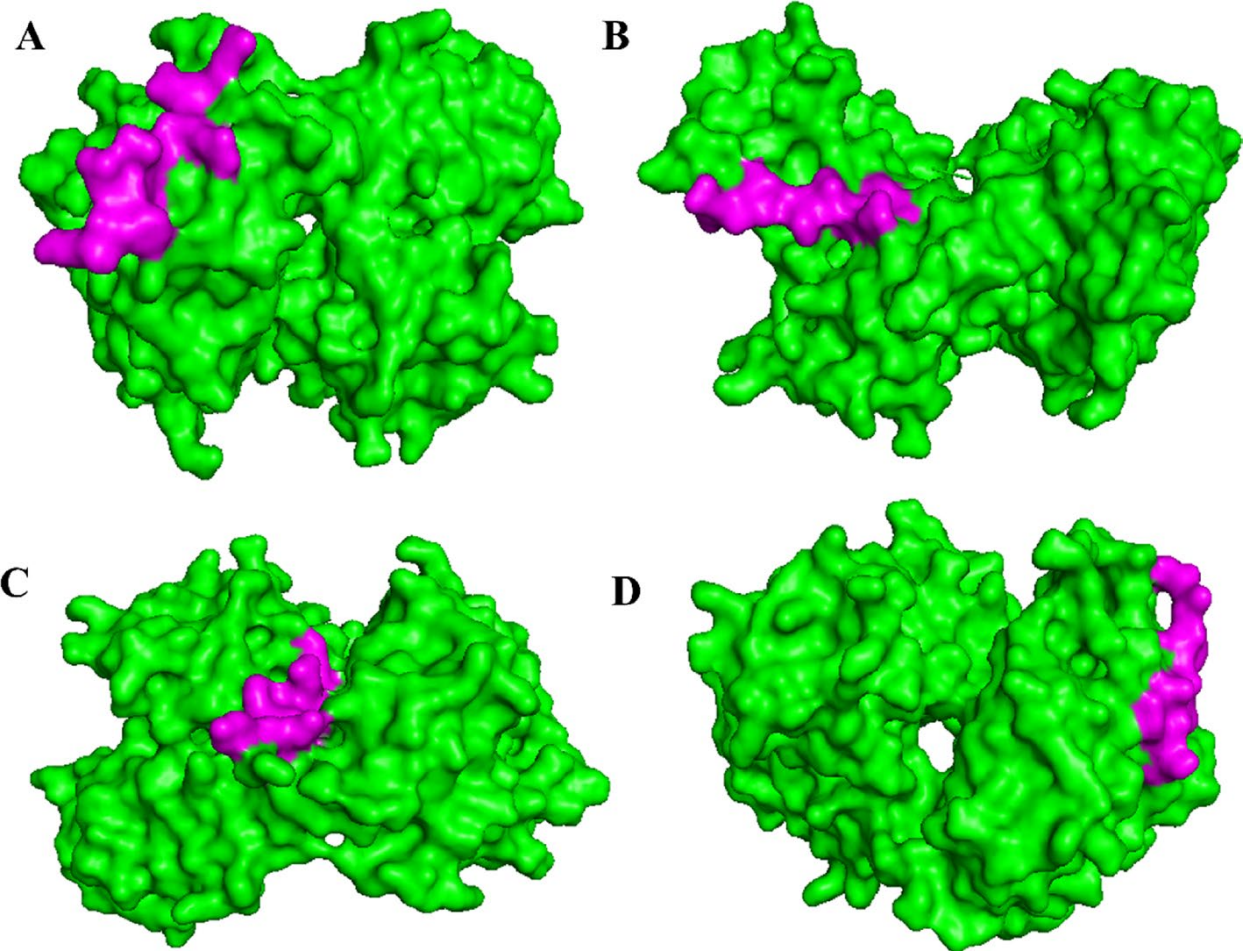
The MEV‐ HLA‐A68 (PDB ID: 4hwz) docking complex, where HLA‐A68 (Receptor) is shown in Green while the pink color represents the peptide‐epitopes as a ligand in the complex procured from the HDOCK server.

For a vaccine to elicit the appropriate immunological responses, it must demonstrate a strong binding affinity with the host's immune receptors. PEP‐FOLD3 was employed to generate the 3D structure of epitopes, utilizing 3D representations of HLAs from the PDB. Peptide binding to MHC complexes was evaluated through HLA‐peptide docking using the HDOCK server. The MM‐GBSA approach, integrating solvent‐scale techniques with molecular mechanical energies, was then applied to assess the binding energies of molecular interactions. Based on the total binding free energy from the docking process, epitopes for MHC‐1 and MHC‐2 in complex with HLAs were selected. Subsequently, optimal models of the MEV were constructed, refined, and subjected to docking with MEV‐HLA‐A68 (PDB ID: 4hwz) as illustrated in Figure 6. The HDOCK server facilitated the docking of the MEV with TLR‐4. The four best peptide epitope docked scores with HLA‐A68 were −18.35, −12.47, −11.05, −35.05 kcal/mol, as shown in Figure 6.

**FIGURE 7:**
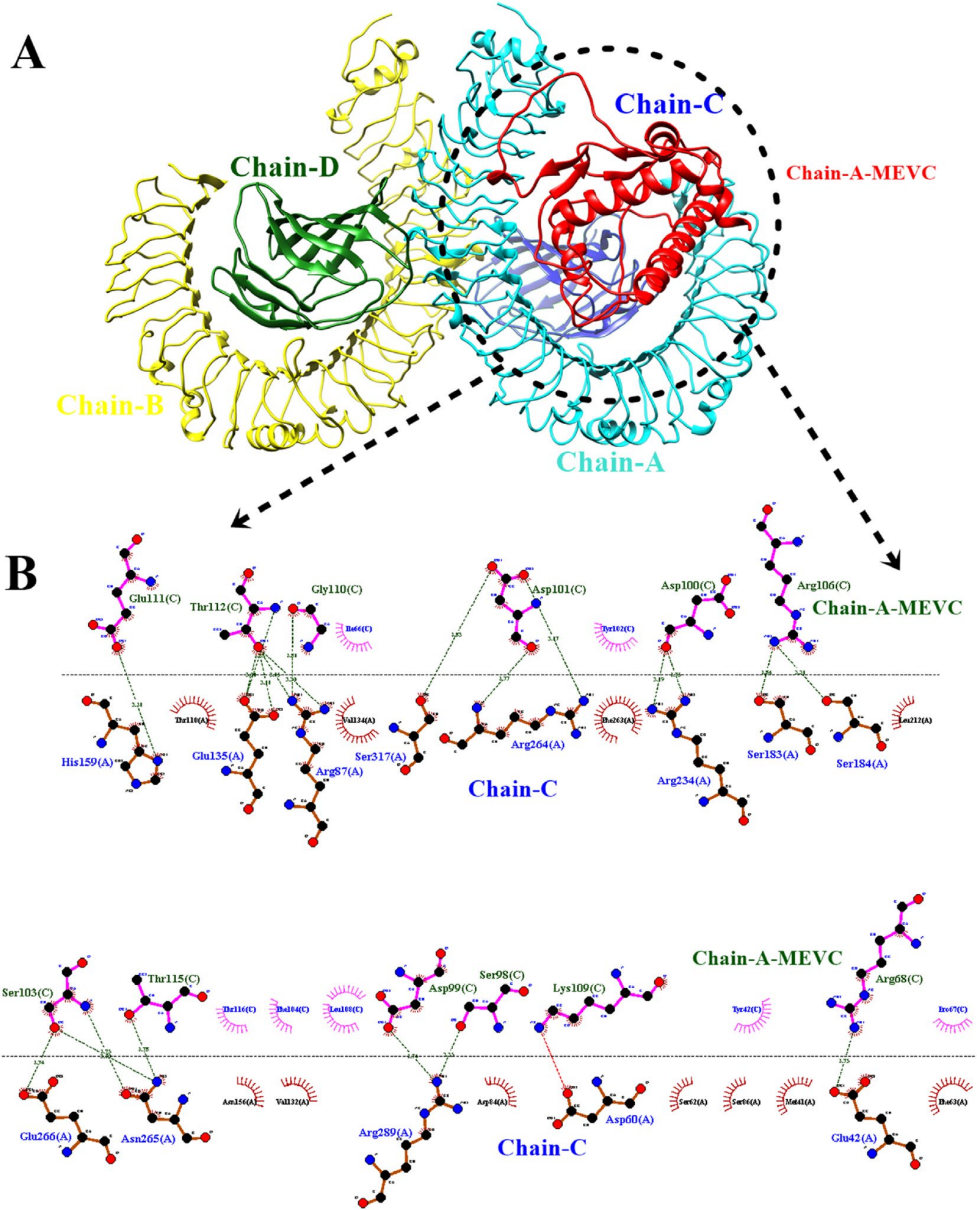
(A) Docked pose of TLR‐4 human receptor protein target with MEVC of PCa. (B) Depicting the hydrogen bonds of the hot spot residues of the chain‐C receptor (blue) with MEVC in (red).

Finally, molecular docking of the MEVC was done to check out the binding pose against TLR‐4 receptor target protein with PDB ID: 3fxi. It has been noticed that strong binding interactions have been observed among them with binding affinities of −278 kcal/mol as shown in Figure 7.

### 3.10 Immune Simulations

**FIGURE 8:**
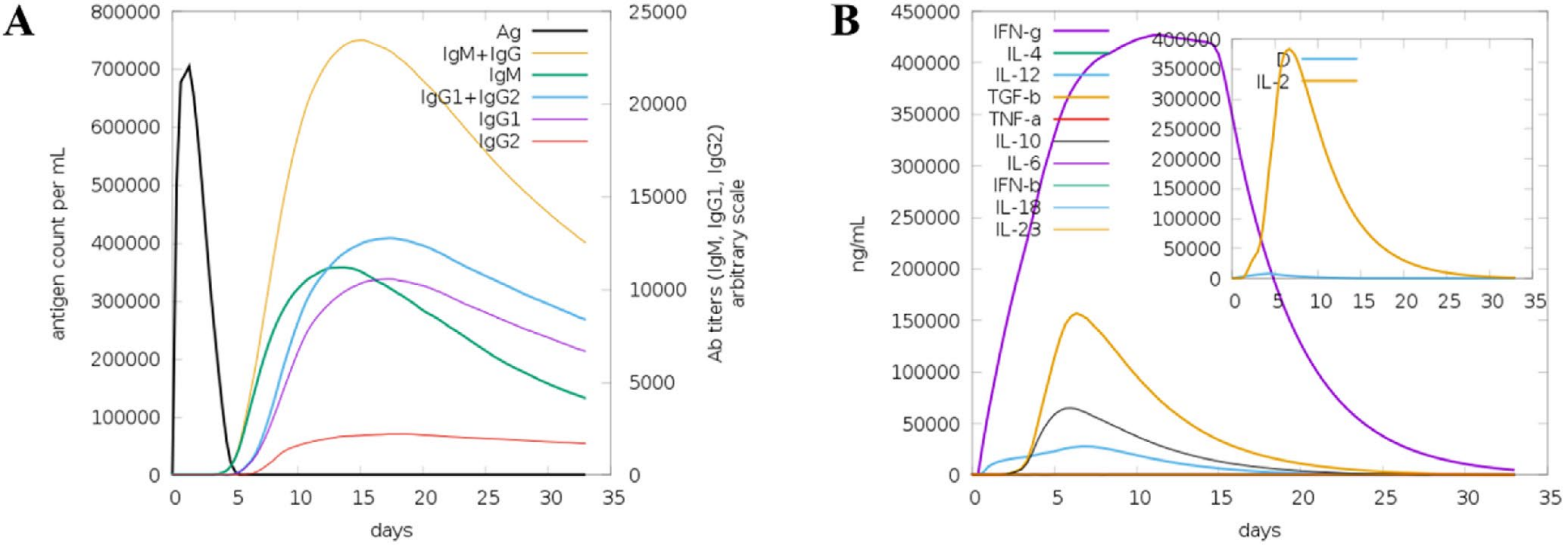
(A) computational immune simulation was conducted to model the host immune system's response to the MEPVC. The (A) panel displays the antibodies, while the (B) panel illustrates the cytokines and interleukins.

To replicate the immune system's reaction to the vaccine, a simulation was conducted with a 4‐week interval between the initial and subsequent doses. The observed immune response in the simulation closely mirrored the actual reactions of the immune system in real‐time (Figure 8). Following the second dose, the immunological response exhibited heightened strength compared to the initial response. Notably, elevated levels of IgM antibodies were observed during the early stages of the immune response. The simulation demonstrated a reduction in antigen levels concurrent with a noticeable increase in various immunoglobulins, including IgM, IgG + IgM, and IgG1 + IgG2 antibodies, during both the initial and subsequent phases of the immunological response (Figure 8A). Robust interleukin and cytokine responses were also evident, with increased quantities of IL‐2 and IFN‐γ in the immune system's reaction to the vaccine. In addition to the cytokine and interleukin responses, a lower Simpson index (D) was observed, indicating a higher degree of diversity (refer to Figure 8B). The results of the immune simulation indicate the establishment of immune memory and the development of immunity against PGH2.

### 3.11 Plasmid Construct With In Silico Cloning

**FIGURE 9:**
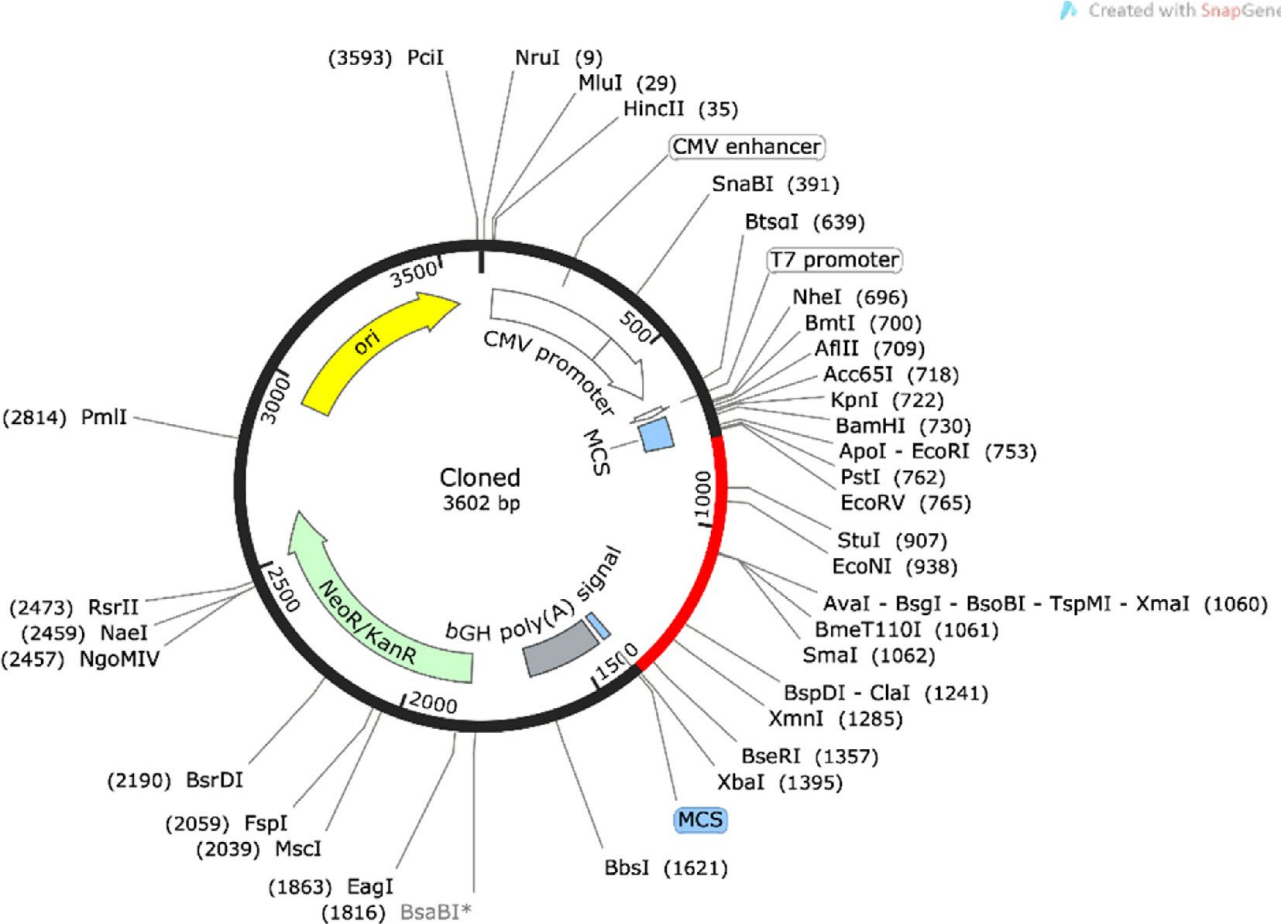
Depicting the pVAX1 vector with a cloned fragment of subunit vaccine in red inserted among restriction sites i.e., NotI and XhoI.

To facilitate its expression in the *E. coli* K‐12 system, in silico cloning was done into a pVAX1 vector. Before this step, a reverse translation of the vaccine amino acid sequence was achieved and optimizing it specifically for *E. coli* competent cells. This strategic adaptation ensures robust vaccine expression during experimental studies. Thus, the JCat server for codon optimization resulted in a CAI value of 0.93 and a GC content of 56.77%. These values align with molecular biology standards for efficient protein expression, indicating proficient vaccine performance within the *E. coli* system. The cloned vaccine is visually represented in red color within the pVAX1 vector (see Figure 9).

### 3.12 Molecular Dynamic Simulation

**FIGURE 10:**
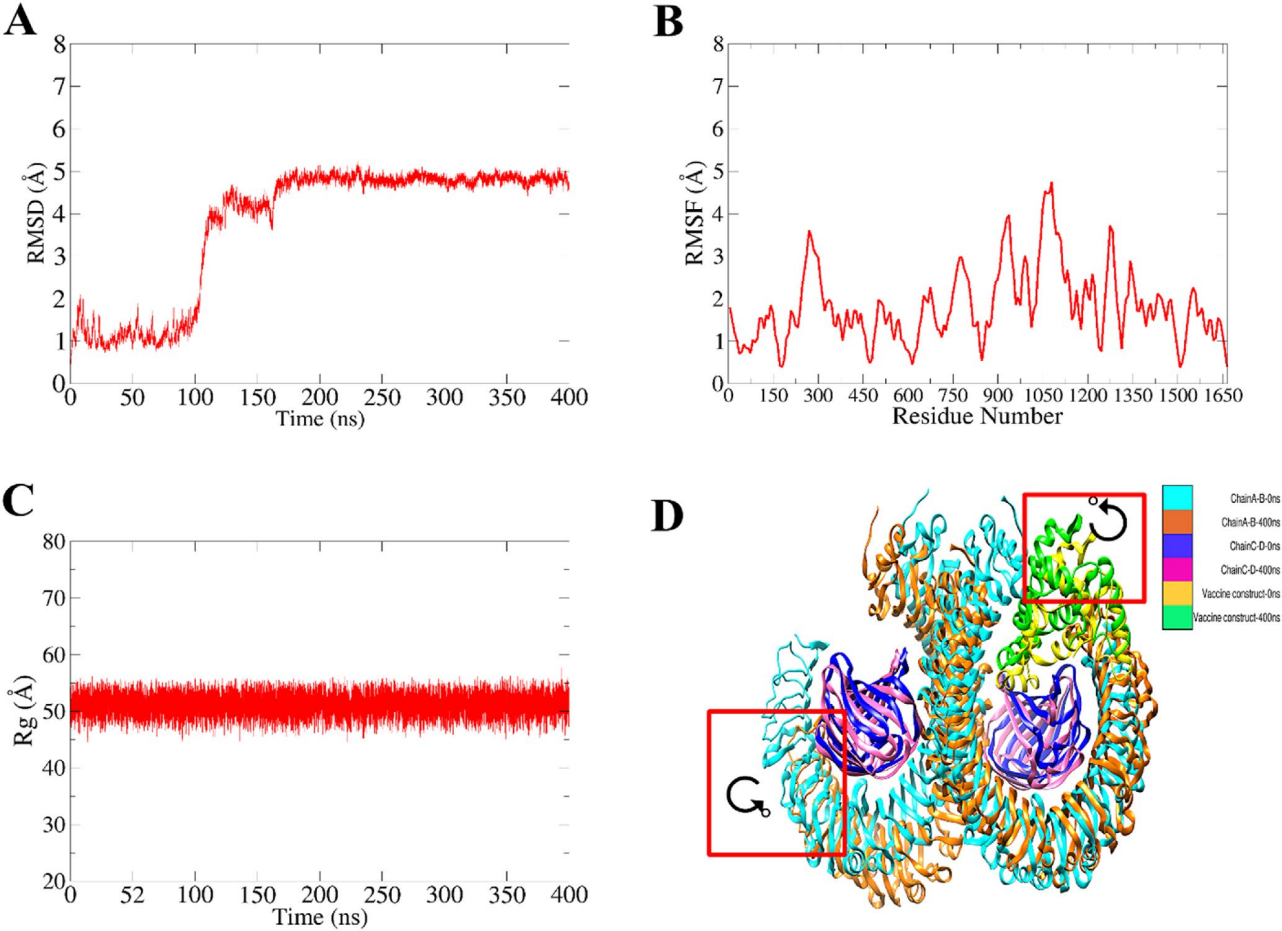
Depicting the dynamics of a simulated complex system. (A) Depicting the RMSD, (B) Presenting the RMSF, (C) Showing the β‐factor, and (D) Showcasing trajectories analysis of the whole simulated complex system at initial time intervals of 0 ns and final time intervals of 400 ns.

The stability of the Multi‐Epitopic Peptide Vaccine Candidate (MEPVC) with Toll‐like Receptor 4 (TLR4) was subjected to in‐depth investigation through MD simulations. Trajectories obtained from these simulations were subjected to crucial statistical analyses to decipher backbone stability and residue flexibility. First, the average distance between backbone carbon alpha atoms in overlaid frames was determined using RMSD (Figure 10A). The system's total RMSD was 3.23 Å, peaking at 5.0 Å at the 100 ns time interval. Potential modifications performed by the complex in response to dynamic forces and impacts from the environment are presented by an initial sudden shift in RMSD up to 400 ns. The system thereafter showed remarkable stability, with no appreciable local or global conformational changes found. After that, the system trajectories were subjected to RMSF analysis (Figure 10B). From their mean locations, RMSF gives information about the mean residual movement of complex residues. The MEPVC‐TLR4 complex's mean RMSF was determined to be 1.80 Å, with a high of 5.0 Å noted at the MEPVC's N‐terminal. Most of the interaction residues between TLR4 and MEPVC showed little variation, suggesting a complex with excellent stability. Finally, *R*_g_ analysis was used to evaluate the compactness of the system. *R*_g_ values show the degree of packing tightness and system compactness, regardless of how high or low they are. This analysis helps determine the overall orderliness of the system. The mean *R*_g_ for our system was 55.8 Å (Figure 10C), indicating a highly ordered and compact nature of the MEPVC‐TLR4 complex, suggestive of system stability. Finally, the trajectories analysis of the whole simulated system has been inferred by superimposing the two simulated time intervals 0 and 400 ns. It has been shown in (Figure 10D) that the system remains static with some angular displacement of structural chain as highlighted in red square.

### 3.13 Binding Free Energies Calculations for the Simulated System

Both the Poisson–Boltzmann (PB) and Generalized Born (GB) models show the net free energy of binding (ΔTOTAL), which indicates that the MEPVC‐TLR4 complex is in a favorable state in pure water. −147.01 and −150.58 kcal/mol are the computed net GB and PB energies for the MEPVC‐TLR4 complex, respectively. It is noteworthy that the gas phase energy (Δ*G* gas) contributes significantly to this total energy, while the solvation energy (Δ*G* solv) has less effect. The gas energy of the system, represented by Δ*G*, in the GB model, is −175.65 kcal/mol, which is the same as in the PB model. Estimated by the MM force field in both techniques, the electrostatic contribution to the system is −65.30 kcal/mol, strongly favoring the net energy. Similar to this, it is stated that MM's van der Waals contribution to the system's stability (−110.35 kcal/mol) is substantial. Table 3 presents the MEPVC‐TLR4 complex net energy along with specific individual binding free energy values.

**TABLE 3:** Binding free energy values for the MEPVC‐TLR4 complex.

Parameter	Vaccine‐TLR‐4 complex
MMGBSA	
Van der Waals energy term	−110.35
Electrostatic energy term	−65.30
Gas phase energy term	−175.65
Solvation energy term	28.64
Net energy term	−147.01
MMPBSA	
Van der Waals energy term	−110.35
Electrostatic energy term	−65.30
Gas phase energy term	−175.65
Solvation energy term	25.07
Net energy term	−150.58

### 3.14 Interactive Analysis

**FIGURE 11:**
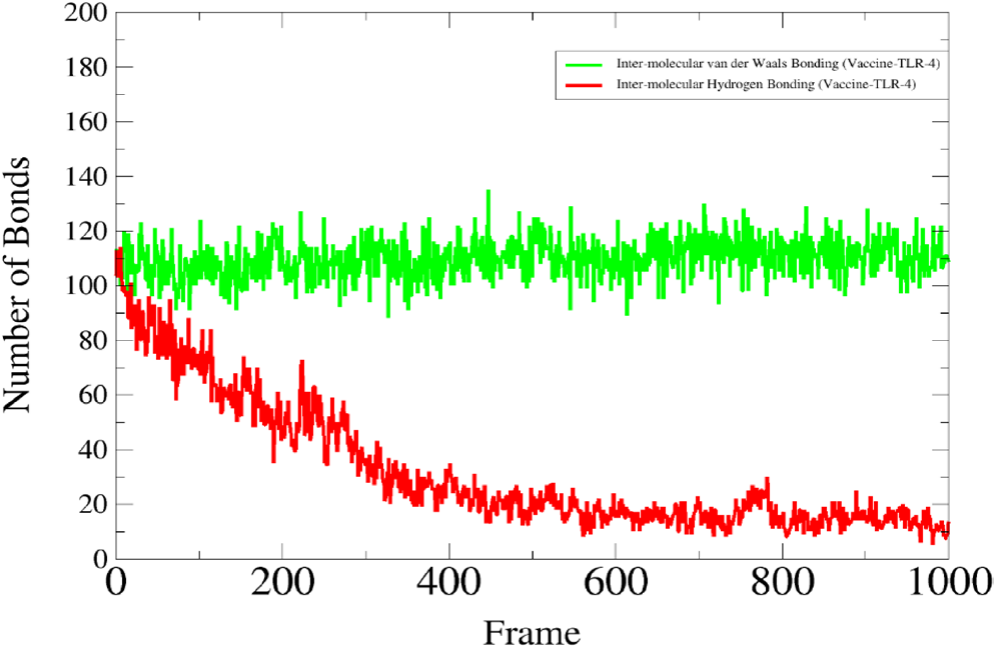
Depicting the hydrogen bonds and van der Waals interactions of the complex simulated system of the vaccine interacting with the targeting TLR‐4 receptor.

**FIGURE 12:**
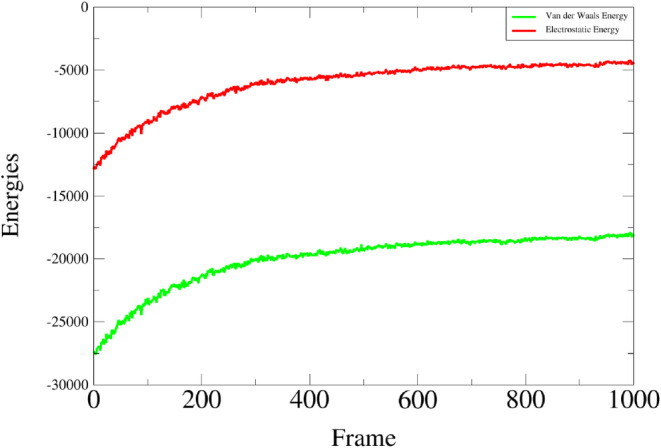
Graphical presentation of van der Waals energies and electrostatic energies of the vaccine construct with TLR‐4.

The interaction analysis specifically covers hydrogen bonding and hydrophobic interaction which are the major interactions in the vaccine‐protein complex. The interaction analysis provides specificity and affinity between the protein and ligand complex [67]. Hydrogen bonding is a key element in molecular recognition and involves a variety of physio‐chemical propositions. The hydrogen bond plot provides information about the intramolecular bonds of a complex during the simulation period. The docked complex shows more resemblance to the target of interest protein TLR‐4. The strength of hydrogen bonds from 0 to 100 ns remains changed during the simulation time as depicted in red color where a high frequency of bonds is observed during the start frames while remaining lower down but static till the end of the simulation (Figure 11). Herein, the van der Waals interaction remains static throughout the simulation time intervals. However, the van der Waals energies distribution started from −27 000 kcal/mol and remained stable by reaching −20 000 kcal/mol whereas, van der Waals binding energies were observed among −12 000 to −5000 kcal/mol as shown in Figure 12.

### 3.15 Waterswap Analysis

A waterswap calculation to verify the accuracy of our results and provide assurance of the vaccine's consistent interactions with receptors was applied. Bennetts, thermodynamic integration, and free energy perturbation were the three methods used in the waterswap approach. Strikingly, the energies of all three complexes showed remarkable stability. The waterswap estimates for the TLR‐4‐vaccine complex were free energy perturbation (−49.12 kcal/mol), thermodynamic integration (−48.6 kcal/mol), and Bennetts (−47.77 kcal/mol). Moreover, the TLR‐4‐vaccine complex (46 kcal/mol) had the highest entropy energy values. According to these results, the vaccine attaches to the receptor well and shows limited physical freedom, which aids in the identification and processing of immunological responses by the immune system.

## 4 Discussion

PCa is the most often diagnosed malignancy among males worldwide and ranks as the fifth highest cause of cancer‐related mortality in this demographic. In 2020, there were approximately 1 414 249 new diagnoses and 375 000 yearly fatalities globally attributable to this illness. PCa is among the most often diagnosed malignancies in over 50% of nations (112 out of 185) worldwide [1, 68]. Canine PCa is comparable to human PCa in various ways, making canines a useful animal model for investigating prostatic carcinogenesis. Adenocarcinoma of the prostate is a primarily invasive and insidious cancer that metastasizes to distant organs in both species [69]. Prostaglandins have a role in a variety of physiological and pathological processes. There is evidence that they may contribute to tumor progression. The primary rate‐limiting enzyme in the biosynthetic route of prostaglandins from arachidonic acid is prostaglandin G/H synthase (PGHS), commonly known as cyclooxygenase or COX [70].

The comprehensive exploration of the Prostaglandin G/H synthase 2 (PGH2) protein, undertaken in this study, has yielded crucial insights into its potential as a candidate for vaccine development, particularly in the context of human PCa. The retrieval of PGH2 amino acid sequences from UniProt [16], its substantial length of 604 amino acids, and the observed upregulation in cases of PCa underscore its significance as a target for immunotherapeutic intervention. The antigenicity evaluation using VaxiJen v.2.0 [20] has confirmed the protein's antigenic properties, substantiating its suitability as a vaccine candidate. The subsequent sequence analysis using Blastp revealed no discernible similarity between PGH2 and human proteins, crucial for avoiding potential cross‐reactivity issues. This distinctiveness is a pivotal factor in ensuring the specificity of the designed vaccine. The identification and selection of B‐cell epitopes, involving a consensus approach from multiple prediction servers, have resulted in the identification of four potential B‐cell epitopes within PGH2. The antigenic, nontoxic, and nonallergenic nature of these epitopes, as assessed by VaxiJen v2.0, AlgPred 2.0, and ToxinPred, further support their candidacy for inclusion in a multi‐epitope subunit vaccine. The T‐cell‐mediated immune response is a critical aspect of vaccine efficacy. A strong cellular immunological response to the vaccine may be induced, as evidenced by the discovery of eight T‐cell epitopes that interact with both MHC‐I and MHC‐II classes. These epitopes specifically target the human alleles HLA‐A68:01 and HLA‐DRB115:01. Enhancing the safety and effectiveness profile of the suggested vaccine is the choice of CTL and HTL epitopes having antigenic in nature, nontoxic, and nonallergenic properties. With its 12 common B‐ and T‐cell epitopes connected by a flexible peptide linker, the MEV formulation is an example of a well‐thought‐out strategy. The inclusion of the STING agonist DMXAA [71, 72] as an adjuvant introduces an additional layer of immune stimulation. The potent anti‐tumor and anti‐vascularization properties of DMXAA make it an ideal functional adjuvant for a cancer vaccine. The 3D modeling of the vaccine component using the AlphaFold server provides structural insights. The confidence estimates generated by AlphaFold [73], particularly the pLDDT metric, serve as a reliable indicator of the model's accuracy. The per‐position pLDDT plot further aids in assessing the conformational stability of the models, with model 5 identified as the most optimal. The “PAE” output from AlphaFold offers valuable information on the anticipated positional error, crucial for interpreting the reliability of the predicted structures. The favorable characteristics observed in the PAE plot and the superior positioning of the C‐terminus in models 4 and 5 highlight the robustness of these models. The immune simulations conducted to replicate the immune system's response to the vaccine provide a dynamic perspective. The observed increase in immunoglobulins, cytokines, and interleukins, along with a lower Simpson index (D) indicative of enhanced diversity, suggests the establishment of immune memory and the development of immunity against PGH2. MD simulations further contribute to the understanding of the MEPVC‐TLR4 complex's stability. The RMSD, RMSF, and Rg analyses collectively affirm the overall stability of the complex. The net free energy of binding, evaluated through GB and PB models, underscores the favorable nature of the MEPVC‐TLR4 interaction in a physiological environment.

Thus, the current study opens new avenues toward experimental vaccine development against cancer diseases and may guide experimentalists to selectively test the proposed set of epitopes and vaccine constructs for immune protection. This will save time and resources and may expedite vaccine development against PCa.

## 5 Conclusions

In conclusion, the integrated strategy including antigenicity evaluation, epitope forecasting, structural modeling, and immunological simulations provides a solid basis for the creation of a multi‐epitope subunit vaccine aimed against PGH2 about PCa. This study's results illustrate the biological need for efficient protein expression, underscoring the robust vaccine effectiveness in the *E. coli* system. Significantly elevated expression levels were detected in the pVAX1 vector during in silico cloning. The vaccine provoked substantial interleukin and cytokine responses, characterized by increased levels of IL‐2 and IFN‐γ. Molecular docking demonstrated a strong binding affinity of −278 kcal/mol, accompanied by hydrogen bonding to several residues. Furthermore, the system's overall RMSD reached 3.23 Å, peaking at 5.0 Å at the 100 ns mark, and exhibited stability up to 400 ns, corroborated by consistent RMSF and Rg measurements. The hydrogen bond cloud residues identified as pivotal locations, markedly affecting the binding energies of MMPBSA and MMGBSA via considerable van der Waals interactions. The unique characteristics of the developed vaccine, such as its safety, specificity, and structural stability, highlight its potential as a candidate for further preclinical and clinical studies.

## Author Contributions

A.A. designed and performed this study and wrote the manuscript.

## Conflicts of Interest

The author declares no conflicts of interest.

## Supporting information


**Figure S1.** Depicting the Ramachandran plot inferring the favored, allowed and disallowed region of the MEVC.
**Figure S2.** (A) Illustrating the Disulfide residues with bonds and energies. (B) Depicting the Disulfide regions in yellow of the vaccine construct. (C) 3D model of the vaccine contruct presenting the yellow highlighted disulfide regions.

## Data Availability

The data that supports the findings of this study are available within this article and in its supplementary material.
