# Supplementary material for: An Integrated Approach to Develop a Potent Vaccine Candidate Construct Against Prostate Cancer by Utilizing Machine Learning and Bioinformatics
Source: Cancer Rep (Hoboken). 2024 Dec 9;7(12):e70079. doi: 10.1002/cnr2.70079 (PMC11626413; doi:10.1002/cnr2.70079)
Supplement: Supplementary file 1 — Figure S1. Depicting the Ramachandran plot inferring the favored, allowed and disallowed region of the MEVC. Figure S2. (A) Illustrating the Disulfide residues with bonds and energies. (B) Depicting the Disulfide regions in yellow of the vaccine construct. (C) 3D model of the vaccine contruct presenting the yellow highlighted disulfide regions. [file CNR2-7-e70079-s001.docx]

**Supplementary materials**


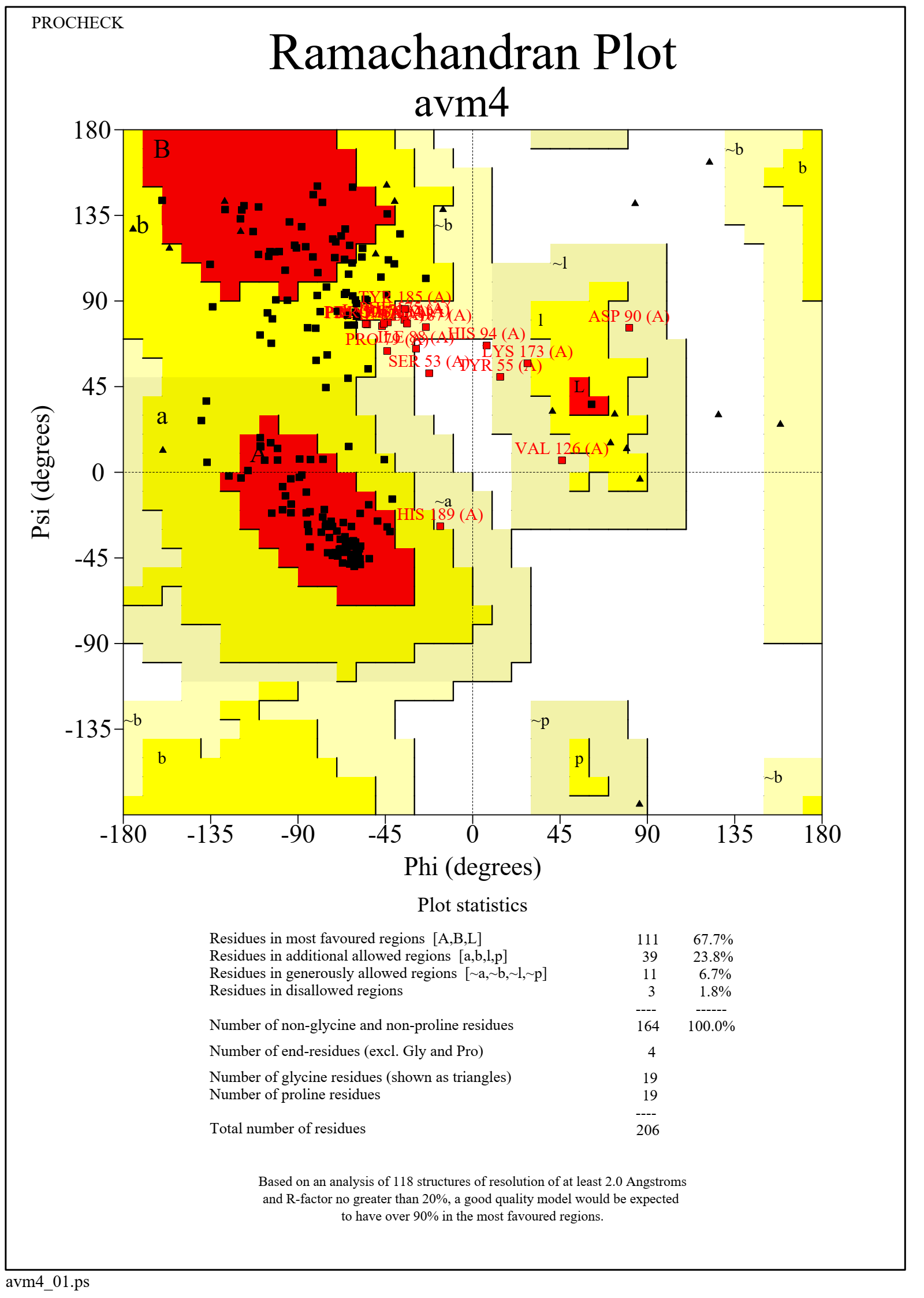


**Figure S1.** Depicting the ramachandran plot inferring the favoured, allowed and disallowed region of the MEVC.


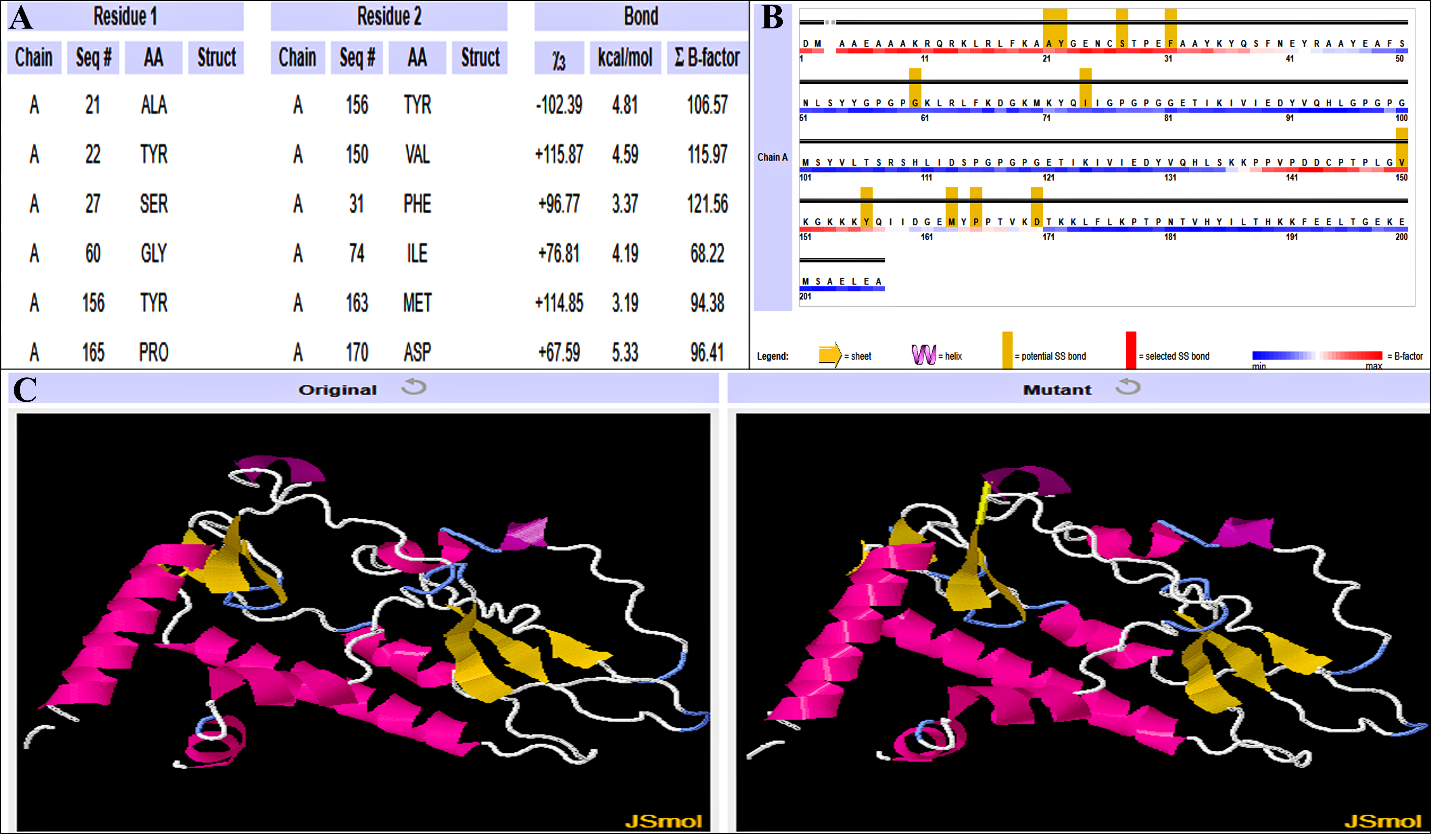


**Figure S2**. (A) Illustrating the Disulfide residues with bonds and energies. (B) Depicting the Disulfide regions in yellow of the vaccine construct. (C) 3D model of the vaccine contruct presenting the yellow highlighted disulfide regions.
